# Inverse Tesla Valve as Micromixer for Water Purification

**DOI:** 10.3390/mi15111371

**Published:** 2024-11-14

**Authors:** Christos Liosis, George Sofiadis, Evangelos Karvelas, Theodoros Karakasidis, Ioannis Sarris

**Affiliations:** 1 Department of Mechanical Engineering, University of West Attica, Egaleo, 12241 Athens, Greece; liosischristos@uniwa.gr (C.L.); gsofiadis@uniwa.gr (G.S.); evangeloskarvelas@gmail.com (E.K.); 2 Condensed Matter Physics Laboratory, Department of Physics, University of Thessaly, 35100 Lamia, Greece; thkarak@uth.gr

**Keywords:** micromixer, Tesla, Fe_3_O_4_, nanoparticles, OpenFoam

## Abstract

Contaminated water has remained an unsolved problem for decades, particularly when the contamination derived from heavy metals. A possible solution is to mix the contaminated water with magnetic nanoparticles so that an adsorption process can take place. In that frame, Tesla valve micromixer and ${\mathrm{Fe}}_{3}O_{4}$ magnetic nanoparticles were selected to perform simulations for encounter maximum mixing efficiency. These simulations focus on inlet velocities ratios between contaminated water and nanoparticles and inlet rates of nanoparticles. The maximum mixing efficiency was 44% for the inverse double Tesla micromixer found for the combination of ${\mathrm{Fe}}_{3}O_{4}$ nanoparticles as the inlet rate and with inlet velocity ratios of $\frac{V_{p}}{V_{c}}=10$.

## 1. Introduction

Water pollutants can contaminate water through natural processes and/or anthropogenic activities. Unlike organic contaminants, heavy metals are not biodegradable and tend to accumulate in organisms [1]. Based on density, metals that exceed 5 ${g/\mathrm{cm}}^{3}$ could be defined as heavy metals. Moreover, heavy metals are also classified into essential (Zn, Cu, Fe, and Co) and nonessential (Cd, Hg, As, and Cr) based on their toxicity [2]. The variety of health problems caused by heavy metals is represented in Table 1.

The necessity of a solution for water purification from heavy metals is more than obvious. Thus, the main problem is to capture the heavy metals and separate them from contaminated water. The procedure that precedes this and is required for capturing the heavy metal ions is to achieve a high mixing performance for the adsorbants. A combination of micromixers and nanotechnology is proposed as a possible solution to the mixing performance, which is expressed with Computational Fluid Dynamics (CFD).

During the last decades, the revolution of nanotechnology has offered possible solutions to an extended variety of problems. Regarding water purification, the idea is to use nanoparticles to capture heavy metal ions. Nanoparticles present advantages, such as a large surface-area-to-volume ratio and catalytic properties. Moreover, magnetic nanoparticles such as ${\mathrm{Fe}}_{3}O_{4}$ offer high magnetic saturation, biocompatibility, and interaction [3], and are insoluble in water [4]. Additionally, they can be separated from the aqueous solution with the use of a magnetic field [5] after the mixing and adsorption process and provide a direct solution to the separation issue. The problem of capturing heavy metals remains, since the magnetic nanoparticles cannot achieve optimal distribution, and thus, the adsorption capacity will not be sufficient. On the other hand, micromixers affect the distribution of the magnetic nanoparticles [6]. The performance of micromixers is highly dependent on the homogenous and efficient mixing of samples [7], which is expressed with the term mixing efficiency.

Micromixers are classified into two categories, i.e., passive [8,9] and active [10,11]. Passive micromixers exploit the micromixer’s geometry to produce complex flow fields for effective mixing [12]. Active micromixers use moving parts and/or external energy to manipulate the fluids [12], also used to improve the mixing efficiency by the disturbance which is produced by external forces [13]. The selectivity between passive and active micromixers is related to geometry and the application. Tesla valves have been used as micromixers for several years; this type of micromixer has plenty advantages, such as a simple structure and a special flow mechanism, good mixing performance for low and high flow rates, and low pressure drop [14]. The term special flow mechanism refers to the ability of the geometry to behave different when the flow is forward or inverse. It causes a higher pressure drop in the reverse direction than in the forward direction [15]. The Tesla valve was selected as a passive micromixer for the nanoparticles distribution; this particular geometry has been used as a micromixer since 2004 [16]. The mixing efficiency of a Tesla valve micromixer is related to the number of valves connected (usually in series)—as the number of Tesla micromixers increases, mixing efficiency is significantly increased and stabilized after several valve units. Other factors that have an impact on mixing efficiency are the contact angle (°), Reynolds number (Re), and the direction of the flow (forward and inverse). Relevant studies have investigated both types of Tesla flow directions, where differences appeared for the same geometry of flow conditions [17]. Further investigations for mixing used numerical and experimental methods for a wide range of flow rates, achieving very good performance. Also, they carried out additional experiments with nanoparticles for biomedical applications [18], since the numerical method does not include nanoparticles.

In the present study, the proposed combination is a micromixer with magnetic ${\mathrm{Fe}}_{3}O_{4}$ nanoparticles. The geometry of the passive micromixer is an inverse Tesla valve, where a heavy-metal-contaminated water stream and a freshwater stream loaded with nanoparticles are inserted in a microfluidic duct with variable inlet velocity ratios and inlet nanoparticles rates. Discrete methods are used to simulate the nanoparticle trajectories and their distribution inside the double Tesla valve geometry in a continuous flow duct. A direct comparison between the forward and inverse Tesla, using the same parameters, boundary conditions, and geometry as the micromixer, is the main finding of the present work. The two types of Tesla valves share the same geometry, but they have different functionalities due to flow phenomena inside the micromixer.

## 2. Materials and Methods

**Figure 1:**
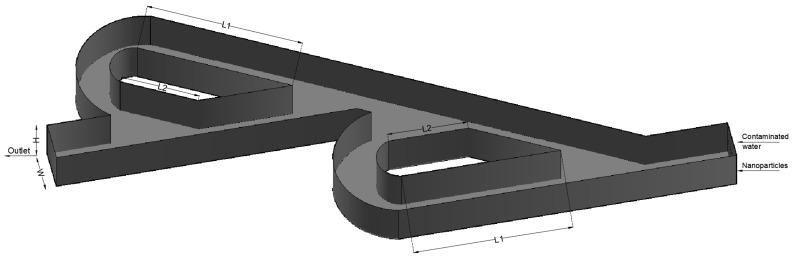
Double Tesla valve geometry.

The simulations focus on the optimum mixing efficiency, and for that reason, both characteristics of the geometry and the nanoparticles were fully defined from related works. The Tesla micromixer geometry uses two units of valves that are connected in series, where the inlet and the outlet of the micromixer were a squared cross-section with height and width of $W=H=10^{-4}$ m, as in our previous work [19]. A length ratio of $\frac{L_{1}}{L_{2}}=\frac{375\;\mu m}{187.5\;\mu m}=2$ was selected from an existing Tesla structure [20]. The two water streams enter the micromixer from different inlets (with equal area), are mixed, and then leave the domain from the common outlet, as shown in Figure 1. This configuration characterizes the flow as inverse. The design of a Tesla valve has no moving parts, does not require input energy, and only uses a spatial structure to push or suppress fluid flow [21]. That allows the fluid to flow unimpeded in one direction (forward flow), but in the other direction (inverse flow), the fluid is blocked. Nikola Tesla claimed that “the resistance in the reverse may be 200 times that in the forward direction. Owing to this, a comparatively very small number of buckets or elements is required for checking the fluid. To give a concrete idea, suppose that the leak from the first element is represented by the fraction $\frac{1}{x}$, then after the *n*th bucket is traversed, only a quantity $n\frac{1}{x}$ will escape and it is evident that *x* need not be a large number to secure a nearly perfect valvular action”. In our study, with only two Tesla units, the flow of the fluid will not be blocked. The equilibrium time of the adsorption process varies from minutes to hours even for the same magnetic composite of iron oxide, for different heavy metal ions. Additionally, the same variety occurs for the adsorption capacity. The adsorption capacity with the fastest equilibrium for each heavy metal ion is represented in Table 2. The range of equilibrium time is 0.5 min up to 15 min, where the time set the minimum combination of micromixer length and the fluid velocity. The mixing length of the Tesla micromixer is difficult to calculate, due to the multiple paths the nanoparticles can follow. In each path, the velocity of the fluid may be different, as the total length varies among the paths. In our study, with the two Tesla valve units, the total length of the micromixer and the velocity of the fluid do not require the minimum combination, but the increase in Tesla units would provide an increase in mixing efficiency and mixing time, according to the literature. In the Results section, an estimation of mixing time for the different inlet velocity ratios is described.

Generally, the adsorption process is based on various factors of the nanoparticles and the aqua solution characteristics. Some of the nanoparticles’ characteristics are size, dispersity, zero point of charge, and colloidal behavior. As concerns the aqua solution, pH and temperature are the two main factors. According to a literature review [2], a specific range of pH and temperature gives maximum adsorption capacity. Moreover, these factors do not affect directly the mixing efficiency, so they are not embedded in the simulations. In conclusion, the benefit of using inverse instead of forward flow for the same number of valves is the increased time of mixing, which is related to the adsorption process.

The majority of existing research has focused on the magnetic iron oxide nanoparticles due to their paramagnetic behavior, high corrosion resistance, and low toxicity [2,29]. Successful removal of heavy metal ions was achieved within 1 min for monodisperse ${\mathrm{Fe}}_{3}O_{4}$ magnetic nanoparticles having a mean diameter size of 13.5 nm [30]. Hence, the ${\mathrm{Fe}}_{3}O_{4}$ spherical nanoparticles with a diameter of 13.5 nm were selected for the particular simulations, with fully defined mechanical properties, such as density (5180 $\mathrm{kg}/m^{3}$ [31]), Poisson’s ratio (0.31 [32]), and Young’s modulus ($200\times 10^{9}$ Pa [32]). The characteristics of the water, such as the density and the viscosity, was taken into account. Also, the heavy metals are not embedded in the simulations, since only the motion of the nanoparticles and their mixing efficiency with the selected parameters are examined here.

Since the Tesla valve is used as a passive micromixer, the factors that could affect the mixing efficiency are the inlet velocity ratio ($\frac{V_{p}}{V_{c}}$) and the inlet rates of the nanoparticles, where $V_{p}$ represents the velocity of the fluid with the ${\mathrm{Fe}}_{3}O_{4}$ magnetic nanoparticles and $V_{c}$ the velocity of the contaminated fluid (water). The selection of the ratio is based on our previous work for the same geometry with forward flow [19,33]; more specifically, the velocity ratios which have been simulated were $\frac{V_{p}}{V_{c}}=1$, $\frac{V_{p}}{V_{c}}=10$ and $\frac{V_{p}}{V_{c}}=20$, while the inlet rates which have been simulated were 500 nanoparticles/s, 1000 nanoparticles/s, and 3000 nanoparticles/s [19,33]. The parameters used for the simulations are summarized in Table 3.

The governing Equations (1) and (2) for the fluid phase are based on the incompressible Navier–Stokes equations, which have been solved in an Eulerian frame, where *p* and *u* are the pressure and velocity, respectively, *t* is time, and μ and ρ are the viscosity and the density of the water, respectively:

(1) ∇·u=0

(2) $$\rho \left[\frac{\partial u}{\partial t}+\left(u\cdot \nabla \right)u\right]=-\nabla p+\mu \nabla^{2}u$$

The discrete motion of particles is solved in a Lagrangian frame. The motion equations of each single particle are based on Newton’s law and are given by:

(3) $$m_{i}\frac{\partial u_{i}}{\partial t}=F_{nc,i}+F_{tc,i}+F_{drag,i}+F_{grav,i}$$

(4) $$I_{i}\frac{\partial \omega_{i}}{\partial t}=M_{drag,i}+M_{con,i}$$

where the index *i* is the *i*th nanoparticle, $u_{i}$ and $\omega_{i}$ are its transversal and rotational velocities, respectively, $m_{i}$ is the mass, the mass moment of the inertia matrix is $I_{i}$, the linear accelerations is $\frac{\partial u_{i}}{\partial t}$, the term $\frac{\partial \omega_{i}}{\partial t}$ corresponds to angular accelerations, the forces that have been embedded in the code are $F_{nc,i}$, $F_{tc,i}$, $F_{drag,i}$, which are the normal contact force, tangential contact force, and hydrodynamic drag force, respectively, $F_{grav,i}$ corresponds to the total force of gravity and buoyancy, and $M_{drag,i}$ and $M_{con,i}$ are the drag and contact moments, respectively.

**Figure 2:**
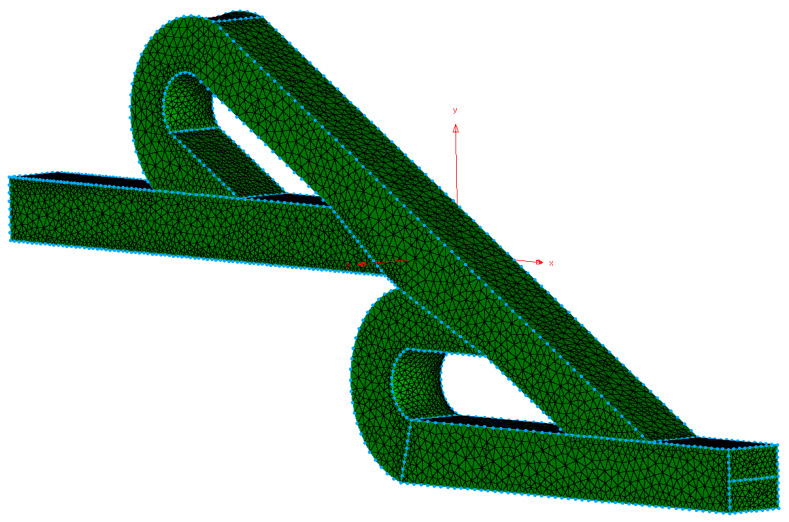
Double Tesla valve mesh.

All the simulations were performed with the same geometry as Figure 1 and with the same unstructured computational grid composed of 107,637 (tetrahedra) cells, as shown in Figure 2. The specific mesh could obtain a significant number of computational predictions in a reasonable time without compromising the validity of the results [34]; a similar mesh was used in our previous work for the forward flow [19].

The mixing efficiency (*n*) is the factor that determines the performance of the micromixer, calculated based on [35,36], where $\sigma_{max}^{2}$ is the square of the maximum possible variance of the tracer concentration and $\sigma^{2}$ is the variance of the concentration species at the selected section. As n reaches 1, the micromixer has optimal performance (fully mixed), while when *n* decreases, the performance decreases as well [36]. In this particular work, the mixing efficiency is evaluated at the exit of the micromixer. That domain separated in N = 16 equal subdomain volumes, where the concentration of ${\mathrm{Fe}}_{3}O_{4}$ nanoparticles at each subdomain was calculated and applied in Equation (5):

(5) $$n=1-\sqrt{\frac{\sigma^{2}}{\sigma_{max}^{2}}}$$

## 3. Results

**Figure 3:**
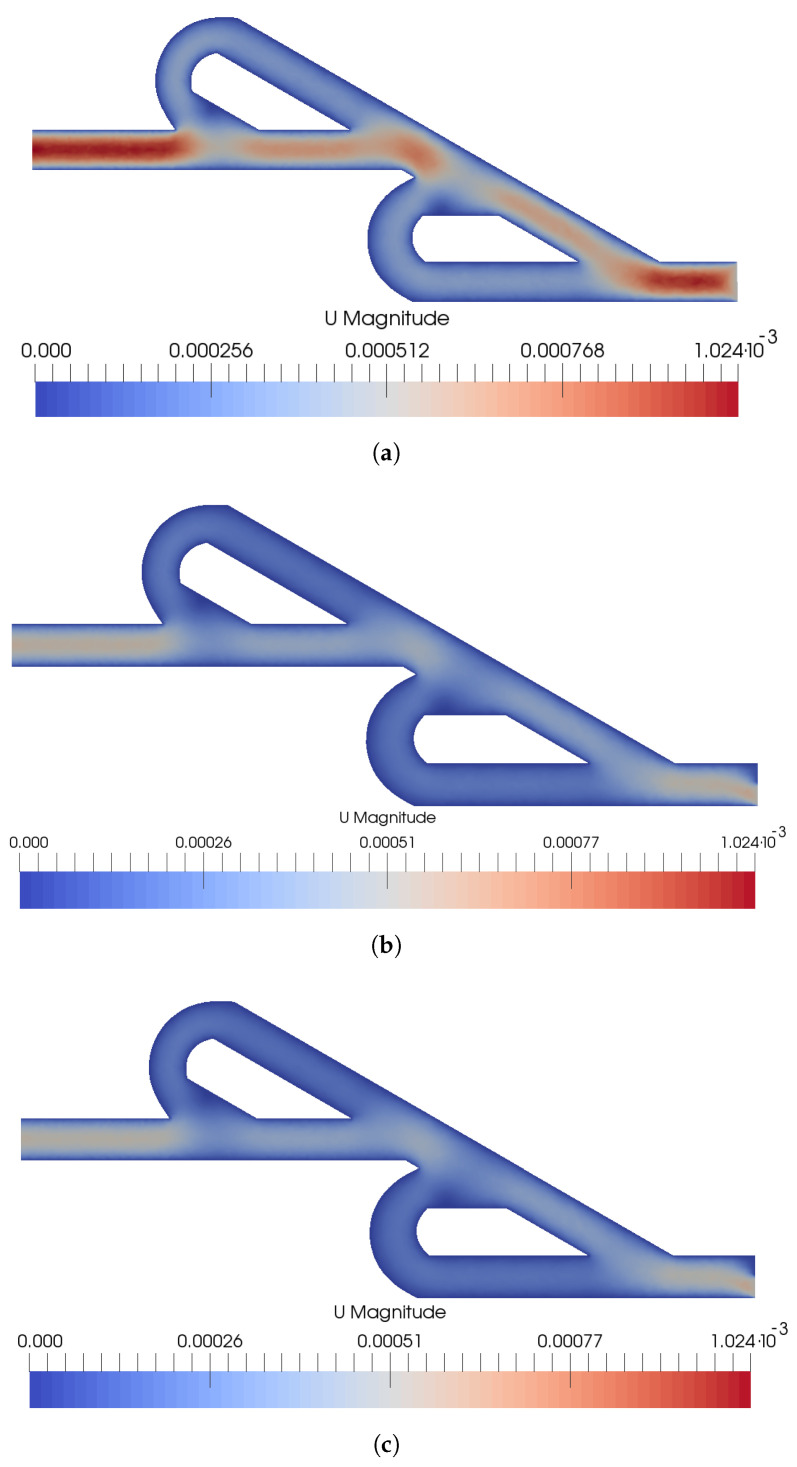
Velocity magnitude for the double Tesla valve micromixer under various inlet velocity ratios (**a**) $\frac{V_{p}}{V_{c}}=1$, (**b**) $\frac{V_{p}}{V_{c}}=10$, (**c**) $\frac{V_{p}}{V_{c}}=20$.

Several simulations were performed with the open source software OpenFoam v.9 for the selected inlet velocity ratios and inlet rates of the nanoparticles. Initially, the velocity field was studied for the post-processing of the simulations to estimate the mixing time of the double Tesla valve micromixer and the Reynolds number (Re). In Figure 3, the maximum velocity inside the micromixer developed for $\frac{V_{p}}{V_{c}}=1$, since as the velocity ratio increases to $\frac{V_{p}}{V_{c}}=10$, the maximum velocity decreases. That decrease of the velocity for the same length of the micromixer leads to an increased mixing time. As the velocity ratio increases to $\frac{V_{p}}{V_{c}}=20$, the maximum velocity decreases further, but not proportionally to the initial ratio transition. Numerically, the maximum velocities inside the micromixer correspond to $1.023\times 10^{-3}$, $6.099\times 10^{-4}$, and $6.065\times 10^{-4}$ m/s for $\frac{V_{p}}{V_{c}}=1$, $\frac{V_{p}}{V_{c}}=10$, and $\frac{V_{p}}{V_{c}}=20$, respectively. Independently from the inlet velocity ratio, the path with the higher mean velocity is the main path and the velocity is minimized at the loop paths.

From the above observations, it is difficult to calculate the mixing time due to the multiple paths that a nanoparticle could follow with different velocities. The assumptions that have been taken into consideration to estimate the mixing time for the selected inlet velocity ratios are that the mixing length is equal to the micromixer length and the maximum velocity exists inside the micromixer. Hence, from these assumptions, the real mixing time will be higher than the estimated mixing time. The estimated mixing time is 1.68 s for $\frac{V_{p}}{V_{c}}=1$, 2.83 s for $\frac{V_{p}}{V_{c}}=10$, and 2.85 s for $\frac{V_{p}}{V_{c}}=20$. According to Table 2, we can estimate the total Tesla valve units which are required for each heavy metal ion to reach the equilibrium. For example, Cu (II) for $\frac{V_{p}}{V_{c}}=1$, $\frac{V_{p}}{V_{c}}=10$, and $\frac{V_{p}}{V_{c}}=20$ required 18, 11, and 11 Tesla valves to achieve the equilibrium, respectively. As mentioned before, at the inverse Tesla valve, the velocity decreases after the addition of the Tesla valve, and thus, fewer Tesla units are going to be used compared to the estimated number.

**Figure 4:**
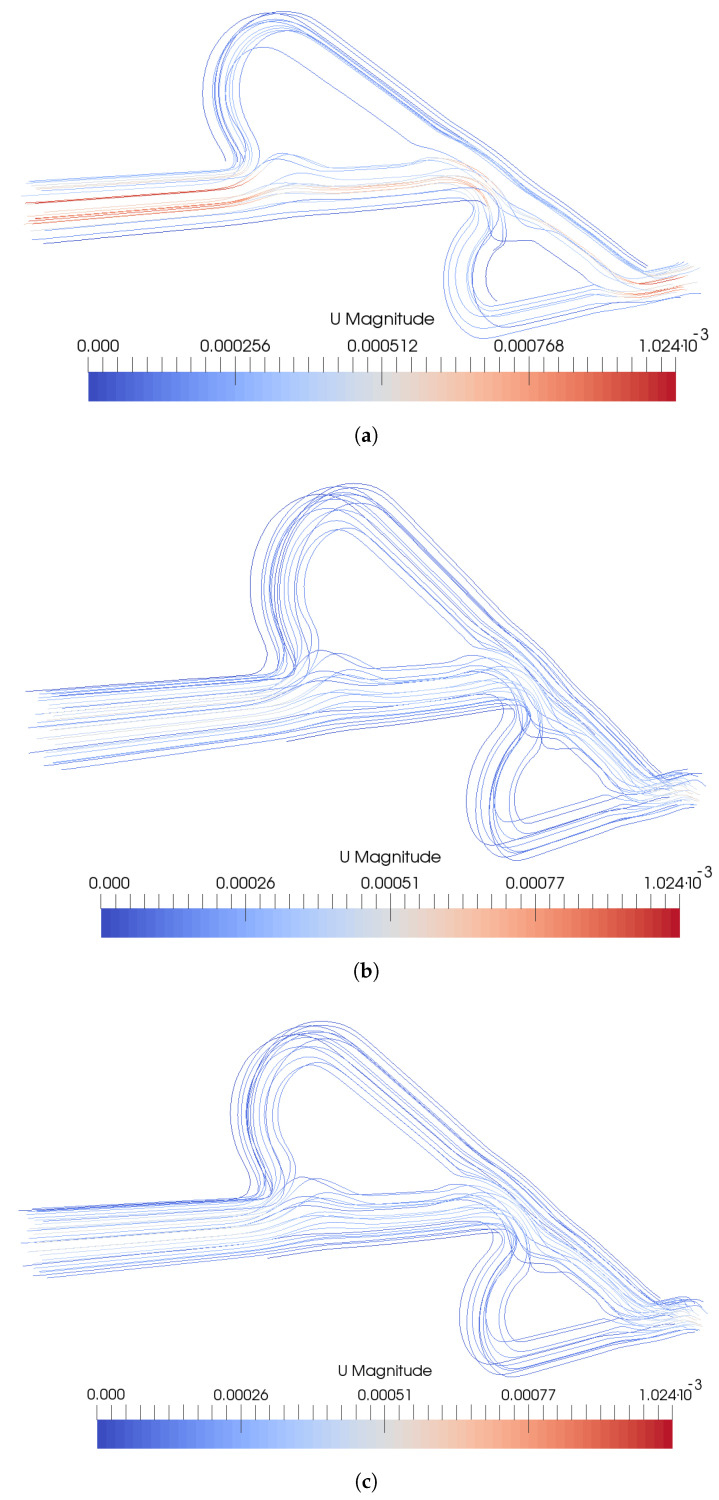
Streamlines under various inlet velocity ratios (**a**) $\frac{V_{p}}{V_{c}}=1$, (**b**) $\frac{V_{p}}{V_{c}}=10$, (**c**) $\frac{V_{p}}{V_{c}}=20$.

Additionally, the streamlines of the velocity magnitude are presented in Figure 4. For $\frac{V_{p}}{V_{c}}=1$, at the main path, higher velocity is observed, which seems to divide the path into two paths, where at the center of the micromixer, the velocity is higher. As the path reaches the walls of the micromixer, the velocity is minimized. This phenomenon is not too intense for the other two inlet ratios. Additionally, the comparison between $\frac{V_{p}}{V_{c}}=10$ and $\frac{V_{p}}{V_{c}}=20$ is difficult from the streamline graphs.

**Figure 5:**
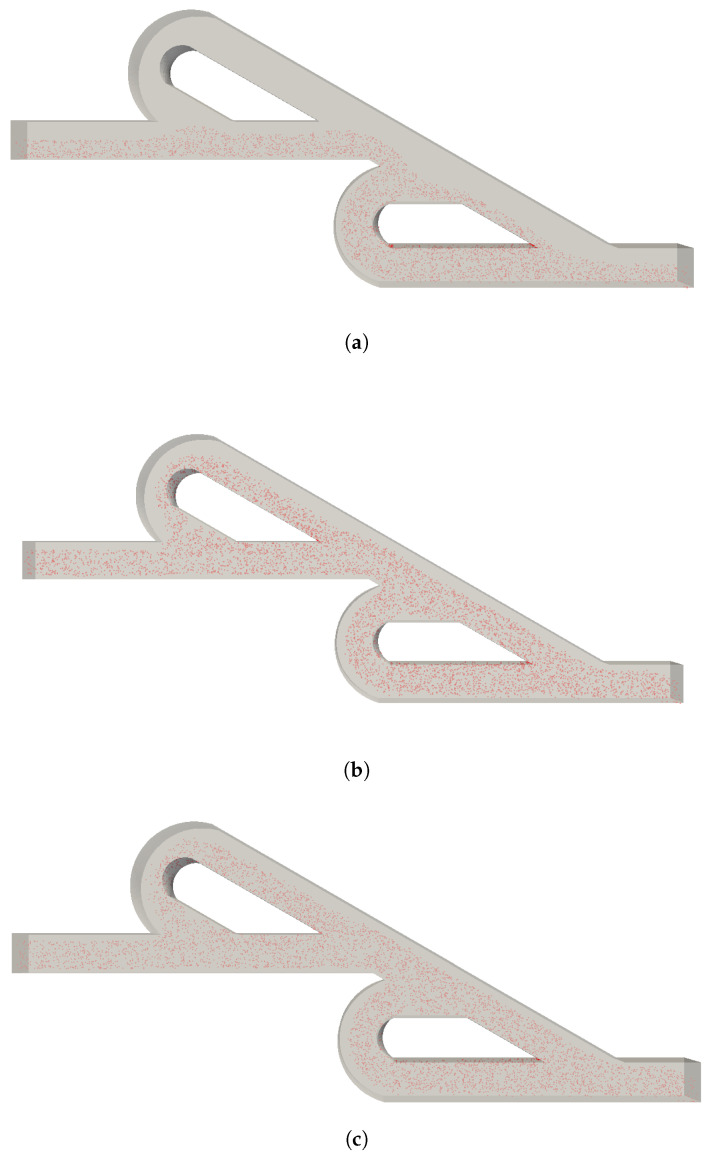
Nanoparticles distribution under various inlet velocity ratios (**a**) $\frac{V_{p}}{V_{c}}=1$, (**b**) $\frac{V_{p}}{V_{c}}=10$, (**c**) $\frac{V_{p}}{V_{c}}=20$ with 500 nanoparticles/s inlet rate.

The particle distribution is shown in Figure 5 for the selected velocities ratios when the inlet rate of nanoparticles was 500 nanoparticles/s. It should be noted that for the other inlet rates of nanoparticles, the results were similar. Firstly, for $\frac{V_{p}}{V_{c}}=1$, the nanoparticles are not distributed uniformly inside the micromixer, and the two inlet streams do not seem to interact, since the nanoparticles are located at the bottom half of the micromixer, as in the initial conditions. Thus, the case with $\frac{V_{p}}{V_{c}}=1$ will not be investigated any further. As the velocity ratio increases to $\frac{V_{p}}{V_{c}}=10$ and $\frac{V_{p}}{V_{c}}=20$, the nanoparticles are located at the main path and the two loops. They are spread across the width of the micromixer, but they are not placed near the walls of the micromixer. Thus, the nanoparticles and the contaminated water will not be fully mixed. Additionally, the design of the Tesla valve and the selection of the inlet ratio affect the optimum and rapid distribution of the nanoparticles at the inlet of the valve, exploiting the entire length of the micromixer. The nanoparticles are located at the loops where the fluid velocity is lower, and thus, the mixing time will be increased. The comparison between $\frac{V_{p}}{V_{c}}=10$ and $\frac{V_{p}}{V_{c}}=20$ could only could be made statistically, since from Figure 5, further outcomes are not clear.

**Figure 6:**
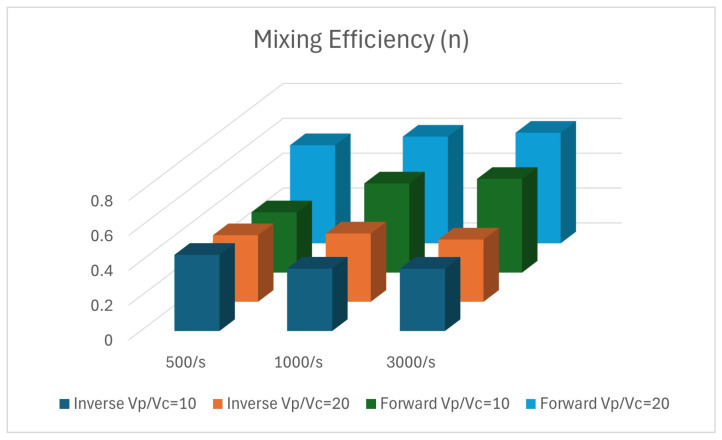
Mixing efficiency (n) for various inlet velocity ratios and inlet rates of nanoparticles for forward and inverse Tesla valve micromixers.

The statistical analysis of the simulations is presented in Figure 6 and performed with the software ParaView v.5.10. The vertical axis represents the mixing efficiency and the horizontal axis shows the parameters that have been studied (inlet velocity ratio and inlet rates of nanoparticles). When the inlet rate was 500 nanoparticles/s, the mixing efficiency was better for $\frac{V_{p}}{V_{c}}=10$ than $\frac{V_{p}}{V_{c}}=20$; numerically, the mixing efficiency was calculated, and found to be 0.44 (44%) and 0.38 (38%), respectively. With the increase of the inlet rate to 1000 nanoparticles/s, we found that better mixing is achieved with a higher velocity ratio. For $\frac{V_{p}}{V_{c}}=20$, the mixing efficiency for the inverse flow was 0.39 (39%), while for $\frac{V_{p}}{V_{c}}=10$, the mixing efficiency was 0.36 (36%). Finally, for the last two cases for the selected inlet rate of ${\mathrm{Fe}}_{3}O_{4}$ nanoparticles, the mixing efficiency was found to be equal to 0.35 (35%) for both cases.

For $\frac{V_{p}}{V_{c}}=10$, the mixing efficiency decreased with the increase of the inlet rates of ${\mathrm{Fe}}_{3}O_{4}$ nanoparticles. On the other hand, for $\frac{V_{p}}{V_{c}}=20$, the mixing efficiency shows unpredictability with the increase of the inlet rate. The flow mechanism inside the geometry affects the mixing performance, as occurred during the mixing efficiency comparison between inverse and forward flow [19], presented in Figure 6.

## 4. Discussion

After the statistical analysis, it arose that the inlet rate of magnetic nanoparticles has a role in mixing efficiency. For $\frac{V_{p}}{V_{c}}=20$, the mixing efficiency range is 35% to 38% for the various inlet rates, while for $\frac{V_{p}}{V_{c}}=20$, this range was 35% to 44%. Additionally, for all cases, the nanoparticles distributed well at the center of the micromixer, but not close to the walls. That phenomenon led to a decrease in mixing efficiency with an increase of the nanoparticles inlet rate due to the increase in the nanoparticle concentration. A direct comparison between the forward and the inverse double Tesla micromixer is necessary. In our previous work with the forward flow, the maximum efficiency reached up to 63% for $\frac{V_{p}}{V_{c}}=20$, while the maximum efficiency for the inverse micromixer was 44% for an inlet velocity ratio equal to $\frac{V_{p}}{V_{c}}=10$. This specific geometry is based on Weng’s valve [20], where after the second Tesla valve, the mixing performance was found equal to 51.93%, which is very close to the current results.

Recently, the Tesla valve has attracted more and more researchers, who have investigated the valve geometry. The variety of geometries and parameters is enormous, but the micromixer is characterized by mixing efficiency. The difficulty to compare mixing performance between other studies and this work arises from the number of Tesla units, which are usually more than two. Moreover, the mixing efficiency is calculated at the exit of the micromixer and not after each unit, and thus, relevant studies do not provide information about the mixing efficiency after the second Tesla valve. Indicatively, a T-junction inlet with six serial valves achieves a mixing efficiency equal to 70.02% [37]. For a Y-junction inlet with 20 serial valves, the mixing efficiency (97%) was found to be 78% [38]. Finally, according to the literature, the highest mixing efficiency was performed for a T-junction inlet with 10 unit pairs [39]. Thus, a mixing efficiency comparison between the present micromixer and previous relevant works shows that the inverse double Tesla micromixer achieves lower performance. However, in order to have reliable results, we will have to compare them using the same parameters. This is very difficult, because the comparison should be performed with the same number of Tesla units. According to the literature, an increase in Tesla valve units leads to an increase in mixing performance.

## 5. Conclusions

The passive micromixer with two Tesla valves in series was investigated for the micromixing effectiveness of ${\mathrm{Fe}}_{3}O_{4}$ magnetic nanoparticles in order to achieve better conditions for the adsorption process. Among the selected parameters, the maximum mixing efficiency (44%) was achieved with 500 ${\mathrm{Fe}}_{3}O_{4}$ nanoparticles as the inlet rate and an inlet velocity ratio of $\frac{V_{p}}{V_{c}}=10$. The mixing efficiency of the inverse flow is lower than the forward flow, with the same geometry and simulation parameters. Encouraging is the fact that with the inverse flow, fewer Tesla valves are required to achieve the necessary mixing time, which is presented in Table 2, due to the flow mechanism. Additionally, the increase in the Tesla units leads to a mixing efficiency increase, according to the literature. Thus, the question for further investigation is whether one should sacrifice mixing efficiency for mixing time, and what is the number of Tesla’s which could achieve simultaneously satisfying mixing efficiency and mixing time. For the case with $\frac{V_{p}}{V_{c}}=1$, no mixing is achieved; thus, a possible solution is to proceed from a passive to active micromixer, for example with an external magnetic field that will take advantage of the properties of magnetic nanoparticles. Moreover, for $\frac{V_{p}}{V_{c}}=10$ and $\frac{V_{p}}{V_{c}}=20$, an external magnetic field could assist the distribution close to the walls of the micromixer, which could lead to better mixing performance.

Finally, the Tesla micromixer has a plethora of features that demand investigation. Some of that is the mixing performance after adding Tesla units, the inlet configuration, and the distance between heavy metal ions and magnetic nanoparticles. Finally, a model that will simulate not only the motion of nanoparticles and heavy metals, but also the adsorption process will be a holistic frame for water purification. 

## Figures and Tables

**Table 1:** Heavy metal impacts at humans health [2].

Heavy Metal	Health Impact
Arsenic (As)	Skin damage, circulatory system issues, protein coagulation, nerve inflammation, muscle weakness, carcinogenicity
Cadmium (Cd)	Kidney damage, carcinogenicity, DNA damage, gastrointestinal irritation, hyperactivity, renal failure
Chromium (Cr)	Allergic dermatitis, diarrhea, nausea, vomiting, headache, neurotoxicity, kidney and liver damage
Copper (Cu)	Gastrointestinal issues, liver and kidney damage, anorexia, Wilson’s disease
Lead (Pb)	Kidney damage, reduced neural development, carcinogenicity, high blood pressure
Mercury (Hg)	Kidney damage, nervous system damage, carcinogenicity, gingivitis, stomatitis, gastrointestinal issues, abortions
Nickel (Ni)	Allergic dermatitis, nausea, chronic asthma, coughing, carcinogenicity, hair loss
Zinc (Zn)	Depression, lethargy, neurological signs, increased thirst, hyperactivity, physical dysfunction

**Table 2:** Equilibrium and adsorption capacity [2].

Iron Oxide Compound	Heavy Metal Ion	Adsorption Capacity (mg/g)	Time (min)	References
*PPY-* $/{Fe}_{2}O_{3}$	Cr (VI)	209	15	[22]
PEI-graftedmagneticporous	Zn (II)	138.8	10	[23]
${Fe}_{3}O_{4}@{SiO}_{2}-EDTA$	Cd (II)	37.59	10	[24]
M-MIONPs	Hg (II)		4	[25]
${Fe}_{3}O_{4}@CTAB$	As (V)	23.07	2	[26]
${Fe}_{3}O_{4}/MMTNC$	Ni (II)	65.78	2	[27]
${Fe}_{3}O_{4}{@SiO}_{2}-NHMFL$	Pb (II)	150.33	0.5	[28]
${Fe}_{3}O_{4}@{SiO}_{2}-NHMFL$	Cu (II)	70.7	0.5	[28]

**Table 3:** Simulations parameters.

inlet and outlet dimensions (m)	Height (H) = Width (W) = $10^{-4}$	
diameter of ${\mathrm{Fe}}_{3}O_{4}$ nanoparticles (nm)	13.5	
inlet rate of ${\mathrm{Fe}}_{3}O_{4}$ nanoparticles	500/s, 1000/s, 3000/s	
**Boundary Conditions**	**Velocity (m/s) **	**Pressure (Pa)**
velocity (m/s) of contaminated water ($V_{c}$)	$5\times 10^{-4}$, $5\times 10^{-5}$, $25\times 10^{-6}$	zero gradient
velocity (m/s) of water with ${\mathrm{Fe}}_{3}O_{4}$ nanoparticles ($V_{p}$)	$5\times 10^{-4}$	zero gradient
Outlet	zero gradient	0
Walls	0	zero gradient

## Data Availability

The original contributions presented in the study are included in the article, further inquiries can be directed to the corresponding author.

## Abbreviations

The following abbreviations are used in this manuscript:

CFD	Computational Fluid Dynamics
Re	Reynolds number
$V_{p}$	Velocity of water with magnetic nanoparticles
$V_{c}$	Velocity of contaminated water
$\frac{V_{p}}{V_{c}}$	Inlet velocity ratio
$L_{1}/L_{2}$	Length ratio of micromixer
*u*	Velocity
*p*	Pressure
*t*	Time
ν	Kinematic viscosity
μ	viscosity
ρ	density
$u_{i}$	Transversal velocity
$\omega_{i}$	Rotational velocity
$m_{i}$	Mass
$\frac{\partial u_{i}}{\partial t}$	Linear accelerations
$F_{nc,i}$	Normal contact force
$F_{tc,i}$	Tangential contact force
$F_{drag,i}$	Hydrodynamic drag force
$F_{grav,i}$	Gravity and buoyancy force
$I_{i}$	Mass moment of the inertia matrix
$\frac{\partial \omega_{i}}{\partial t}$	Angular accelerations
$M_{drag,i}$	Drag moment
$M_{con,i}$	Contact moment
*n*	Mixing efficiency
$\sigma^{2}$	Concentration variance
$\sigma_{max}^{2}$	Maximum possible variance
